# The Sustainability Journey of Chinese Ready-Meal Companies Going Global: Configurational Analysis Using the Technology-Organization-Environment Framework

**DOI:** 10.3390/foods13142251

**Published:** 2024-07-17

**Authors:** Dengjiecai Zhang, Gaofeng Wang

**Affiliations:** School of Management, Henan University of Technology, Zhengzhou 450001, China; 15228309878@163.com

**Keywords:** pre-cooked meal industry, overseas supply chain sustainability, the technology-organization-environment (TOE) framework, fsQCA, internationalization strategy, environmental, social, and governance (ESG)

## Abstract

In the new wave of globalization, China’s pre-cooked meal industry has rapidly emerged as a significant player in the food supply chain domain, owing to its convenience and diverse choices. Amidst intensifying market competition, crafting a sustainable supply chain for overseas expansion has become an indispensable core element for driving corporate internationalization. This study focuses on the sustainability generation mechanism of the overseas supply chain of Chinese pre-cooked meal A-share listed companies, employing the technology-organization-environment (TOE) framework and fuzzy-set qualitative comparative analysis (fsQCA) method. Herein, an in-depth analysis of 388 data points from 17 listed companies has been conducted. The study systematically reveals the key factors affecting the sustainability of overseas supply chains and identifies three critical sustainability generation paths: the technology-driven and norm compliance model (path 1), the market sensitivity and product innovation model (path 2), and the robust operation and risk control model (path 3). The present study not only introduces a novel perspective on the application of a TOE framework within the realms of sustainable supply chain management and environmental, social, and governance (ESG) criteria but also offers clear directions for companies to enhance sustainability in their overseas expansion process. This includes strengthening technological research and development, compliance construction, market adaptability, product diversity, infrastructure construction, and risk management. Moreover, the research findings provide valuable insights for policymakers in promoting the internationalization of the pre-cooked meal industry and enhancing industry competitiveness.

## 1. Introduction

In the context of globalization, the internationalization of the food supply chain has become an increasingly critical strategy for enterprises to expand their markets [1,2]. The Chinese pre-cooked meal industry, with its unique convenience and product diversity, has rapidly risen and demonstrated significant market potential [3]. However, as competition in both domestic and international markets intensifies, companies must not only focus on economic benefits but also pay attention to the sustainability of their supply chains [4]. Particularly during the process of going global, ensuring the sustainability of the supply chain has become a hot topic of concern for both industry and academia [5]. Especially when caught up in the tide of globalization, food export enterprises must confront the complex challenges of international market uncertainties, cultural differences, and adapting to legal and regulatory frameworks [6]. Moreover, increasing awareness among consumers regarding food safety and health has raised the bar for transparency and traceability within the supply chain [6], along with increasing awareness of food safety and health among consumers, which promotes higher requirements for the transparency and traceability of the supply chain [7]. Therefore, it is particularly urgent to explore the sustainability generation mechanism of overseas supply chains in depth. Chinese pre-cooked meal A-share listed companies, with their market influence, information transparency, industry benchmarking role, policy orientation, capital advantages, and international perspective, naturally become an ideal sample for studying the sustainability generation mechanism of the overseas supply chain.

Despite pioneering studies that have explored various aspects of the prepared food sector, such as consumer behavior and willingness [8,9], the environmental and economic sustainability of fresh-cut and pre-cooked vegetables [10], European consumers’ preferences for cross-border fresh fruits and vegetables [11], the digital transformation within the prepared food industry [12], the establishment of industry standards [13], risk management strategies [14], trade development models, factors influencing the sustainability of cross-border agricultural product supply chains [15], and the sustainable development pathways enabled by cross-border e-commerce platforms for agricultural products [16], there remains uncharted territory in the academic discourse: the sustainability of overseas supply chains for prepared food enterprises. In particular, we still lack a comprehensive understanding of the core factors of supply chain sustainability in the process of enterprises going global, which limits our insight into the successful path of enterprise internationalization. The overseas success configuration path of Chinese A-share listed pre-cooked meal companies is an area that has not been fully explored, which is crucial for revealing the key factor combinations for success. Understanding these factor combinations can not only provide enterprises with a replicable path to success but also help enterprises choose the most suitable internationalization strategy according to their own conditions [17]. Furthermore, the ways in which pre-cooked meal companies can enhance their sustainability performance in overseas markets through optimized supply chain management are of great significance to the long-term competitiveness of enterprises in the global market and the sustainable development of the industry. Enterprises need to enhance their global competitiveness and ensure that their operations meet the requirements of sustainable development through refined strategic and operational adjustments. In terms of the application of theoretical frameworks, the TOE framework, while providing us with a powerful tool for analyzing corporate innovation and change, has not been widely applied in the context of research on supply chain sustainability, limiting our in-depth understanding of industrial changes and dynamics. At the same time, ESG performance is often regarded as a core competitiveness factor of enterprises, an important support for driving enterprises to grow stronger and sustain development [18], and the current research often adopts qualitative descriptive methods [19], which have limitations in revealing the complexity of multiple-factor interaction. The fuzzy-set qualitative comparative analysis (fsQCA) method, as an analytical tool capable of handling multiple conditional variables and identifying the effects of different factor combinations [20], provides us with a new research perspective. It can help us identify various factor combinations that lead to success, thereby providing enterprises with more precise and flexible decision support. Based on this, this study aims to explore the following questions:

**Question 1:** On the basis of TOE theory, what core factors are key to determining the sustainability of enterprises’ overseas supply chains?

**Question 2:** Using the fsQCA method, what are the main configuration paths that support the success of Chinese A-share listed pre-cooked meal companies in overseas markets?

**Question 3:** Based on the analysis results regarding key factors and configuration paths, how should pre-cooked meal companies optimize their supply chain management in a targeted manner to enhance their sustainability performance in the process of going overseas?

This study will apply TOE theory and the fsQCA method to conduct a systematic analysis of the sustainability generation mechanism of the overseas supply chain of Chinese A-share listed pre-cooked meal companies. We hope that through this research, we can deepen our understanding of the factors affecting supply chain sustainability, reveal successful development paths, provide targeted practical guidance for related companies, and further promote the sustainable development of the industry. At the same time, this study will also provide valuable insights for policymakers to assist in the formulation and implementation of relevant policies.

The remaining content structure of this study is as follows: Section 2 will introduce a literature review and related theories, providing a solid theoretical foundation for subsequent research. Section 3 will introduce case selection, data sources, and sample characteristics, and, based on TOE theory, combined with ESG, will build a model for the sustainable generation mechanism of the overseas supply chain of listed pre-cooked meal companies, and will also introduce the fsQCA method and analysis steps. Section 4 will conduct empirical analysis to identify key success factors and their conditional configurations. Section 5 will compare the analysis results with existing theories and findings in the literature, discuss the consistency and differences of the research, and discuss the implications of the research results for practice and policy. Finally, Section 6 will summarize the main findings of the research, point out the contributions and limitations of the research, and propose directions for future research.

## 2. Literature Review and Theoretical Foundation

(1) Literature Review

The pre-cooked meal industry, as an emerging force in the food industry, has rapidly expanded on the global stage in recent years. With its convenience, efficiency, and quality assurance, it has precisely met the strong demand for healthy, convenient, and green-produced food in our modern, fast-paced life [21]. However, China’s pre-cooked meal industry is currently facing challenges such as market immaturity, an incomplete standard system, low industry concentration, and a scarcity of leading enterprises [22]. As companies expand both domestically and internationally, the complexity of supply chain management increases dramatically, especially in terms of ensuring food safety, improving logistics efficiency, and promoting sustainable development. Against the background of globalization, the overseas supply chain management of pre-cooked meal companies shows multi-dimensional challenges and broad opportunities [23].

In an in-depth analysis of supply chain management, Ma Lin [24] has highlighted the urgency of optimizing the entire process from raw material procurement to the final product’s distribution, emphasizing the decisive impact of efficiency improvements in each step on overall supply chain performance. The complexity of the prepared food supply chain demands comprehensive integration, from the initial processing of raw materials to the final consumer market [25], while the potential of digital technology in supply chain management within the current “stay-at-home economy” trend cannot be overlooked [26]. The perfecting of policies and regulations [27], control of data quality [28], enhancement of food safety standards [29], the importance of food safety compliance [30], the resolution of financing difficulties [31], and the emphasis on environmental protection and saving energy [32], as well as meeting the carbon emission policies in the different scenarios of various countries [33], are all key to promoting the sustainable development of the industry. Technological innovation, especially the introduction of blockchain technology [34], and the positive incentive of digital trade on the technological innovation of export enterprises [35] have opened up new paths for the future development of the prepared food industry. The application of system dynamics in risk management [36] and global value-chain positioning [37] provides scientific risk assessment and response strategies for prepared food enterprises. At the same time, by exploring the differences in preferences for prepared foods among consumers in different European countries, the discussion of how cultural factors, purchasing power, and availability affect consumer choices highlights the factors that influence the market strategy of prepared food enterprises when going global [11].

Furthermore, existing research has mainly been conducted from the industry and consumer perspectives, while issues in the overseas supply chain of prepared food enterprises, such as the clarification of industry standards [38], the strengthening of risk management [39] the establishment of supply chain cooperation mechanisms [40], the connection with rural revitalization strategies [41], the exploration of market potential [42], the innovation of international trade models [3], and changes in the external policy environment [43] remain challenges that the prepared food industry must face.

Despite significant achievements in research, there are still limitations. Currently, most studies focus on the domestic market, and further exploration is needed regarding the key factors and their interactive combination effects on the sustainability of overseas supply chains. Moreover, how to enhance the sustainability performance of enterprises in overseas markets through optimized supply chain management is a topic that has not yet received adequate research and understanding. Additionally, the combined application of the TOE theory and fsQCA analysis method is not widely popularized, which limits stakeholders’ in-depth understanding of the industry’s evolutionary mechanisms, especially in identifying and analyzing complex causal relationships.

As a result, taking 17 listed pre-cooked meal companies as examples, this study deeply analyzes the key factors affecting the sustainability of overseas supply chains for pre-cooked meals, based on the TOE theory and fsQCA analysis method. It explores the successful path of sustainable development for listed pre-cooked meal companies’ overseas supply chains, providing insights for enterprises and policymakers to make more precise strategic decisions in the rapidly changing market environment, and prompting the pre-cooked meal industry to move toward a higher level of sustainable development.

(2) Theoretical Foundation

The TOE framework, proposed by Tornatzky and Fleischer, is an empirically validated analytical tool that focuses on an in-depth analysis of technological characteristics, organizational alignment, and the external environment [44]. Its flexibility allows researchers to customize the framework indicators for different fields and objectives [45]. Since its introduction in 1990, the TOE framework has demonstrated strong explanatory power and broad applicability in the analysis of technology adoption, organizational change, and environmental adaptability [46].

In this study, the TOE framework is applied to explore the sustainability generation mechanism of the overseas supply chain of Chinese pre-cooked meal A-share listed companies. This framework provides a macro perspective, enabling us to comprehensively examine the international development of the pre-cooked meal industry and accurately identify the key factors affecting its development. The in-depth analytical capabilities of the TOE framework help us understand the interaction of technological, organizational, and environmental factors in the context of globalization, providing theoretical support for the sustainable development of the pre-cooked meal supply chain and assisting stakeholders in formulating effective strategies.

## 3. Research Method

### 3.1. Data Sources and Case Selection

In this study, we have meticulously designed a sample selection process to ensure the rigor of the research findings and reveal profound insights into the dynamics of the pre-cooked meal market. We prioritized listed companies as the research subjects, based on several core considerations: firstly, the financial transparency and regular reporting requirements of listed companies provide a wealth of reliable data resources for the study; secondly, these companies hold leading positions in the pre-cooked meal industry, and their innovative practices and market performance are crucial for understanding industry trends.

To further ensure the relevance and industry representativeness of the sample, we selected A-share listed companies from the “2023 Top 50 Pre-cooked Meal Leading Enterprises” list released by the China Media Group. This method not only indicates the industry-leading position of the sample companies but also reflects their active role in promoting industry innovation and market development. In addition, we adopted a cross-industry sampling strategy, covering various related fields such as professional pre-cooked meal companies, catering supply chain companies, frozen food companies, and catering companies. This diversified sample composition enhances the universal applicability of the research findings and allows us to explore the similarities and differences in the sustainability generation mechanism of the supply chain against different industry backgrounds. To improve the timeliness of the research and ensure the accuracy of the analysis, we focused on data from 2022. This decision enables this research to reflect the latest market conditions and corporate behavior, providing a reference for the future development trends of the industry. In the process of sample screening and data verification, we implemented strict standards and ultimately determined 17 pre-cooked meal companies as research samples.

### 3.2. Sample Characteristics

The 17 listed companies selected in this study are 70% distributed in coastal areas, with Shandong Province taking the lead, having 5 companies on the list, followed by Henan Province with 4 companies, Fujian Province with 3 companies, Guangdong with 2 companies, Jiangsu with 2 companies, Liaoning with 1 company, and Hebei with 1 company. The representative companies producing pre-cooked meals that are studied in this paper can be roughly divided into four categories: one is companies producing professional pre-cooked meals, such as Wei Zhixiang, Gai Shi Food, etc.; the second is catering supply-chain companies, as represented by Sheng Nong Development, Longda Food, Guolian Aquatic Products, Qian Wei Central Kitchen, etc.; the third is frozen-food companies, as represented by Anjell Food, Sanquan Food, etc.; the fourth is catering companies, such as Guangzhou Restaurant. The specific content is shown in Table 1 as follows.

### 3.3. Construction of the Index System and Variable Measurement

Building upon the TOE theoretical framework, an in-depth analysis of the existing literature, and integration with current industry practices, this study aims to construct a comprehensive index system to assess and promote the sustainability of the overseas supply chain of Chinese pre-cooked meal A-share listed companies. This index system not only covers the key dimensions under the TOE framework but also pays special attention to the specific needs and challenges of the pre-cooked meal industry in international market operations. By thoroughly interpreting the existing theories and closely observing actual business processes, we have identified a series of key indicators that together form the basis for assessing the sustainability of the overseas supply chain of pre-cooked meal companies.

Technical Dimension: This study focuses on examining the impact of innovative production technologies and technological advancements on the internationalization of the prepared food supply chain. Industrialization, scaling, and the standardization of production methods are regarded as the core technological forces propelling prepared food enterprises toward venturing overseas. Investment in research and development (R&D) and the integration of intelligent information technology and automation are identified as key variables; they are the driving forces behind supply chain innovation and operational efficiency. Innovation in R&D can bring new product solutions to meet market demands while enhancing resource utilization efficiency. The application of intelligent information technology can optimize the transparency and responsiveness of the supply chain, reducing operational costs. Therefore, this study selects R&D investment, supply chain transparency, and the integration of intelligent information technology and automation as factors to measure the technological dimension of the overseas supply chain of prepared food enterprises.

(1) R&D Investment

Innovation in products is a vital pathway for companies to enhance their competitiveness and explore international markets. A company’s innovation is often seen as a continuum, with more innovative companies being dedicated to developing more competitive products or services than their competitors [47]. Product innovation can better promote the improvement of a company’s technological knowledge capabilities, and technological knowledge is the core of driving corporate development and gaining a competitive advantage [48]. This study measures the product innovation variable, based on whether the R&D investment of listed pre-cooked meal companies in the 2022 reporting period is higher than the average, and uses the quartile method to calibrate R&D investment.

(2) Supply Chain Transparency

Supply chain transparency refers to the visibility and accessibility of information throughout the entire supply chain. In detail, it involves the information gained at every stage from product production to the final consumer being understood by the relevant stakeholders. It ensures that companies understand the environmental impact of their product production processes, including raw material acquisition, energy use during production, and waste handling. Moreover, transparency enables companies to identify and improve social issues within the supply chain, such as labor conditions, fair pay, and the prohibition of child labor. The establishment of a supply chain traceability system is used to measure the degree of supply chain transparency; if such a system is established, it is assigned a value of 1, otherwise, it is assigned a value of 0.

(3) Intelligent Information Technology and Automation

Intelligent information technology and automation refer to the use of advanced information technology and automation systems to optimize and enhance the efficiency and effectiveness of supply chain management. This includes using data analysis, artificial intelligence, the Internet of Things (IoT), machine learning, automated robots, and automated warehouse management systems to improve the transparency, response speed, and flexibility of the supply chain. It is required that pre-cooked meal companies establish an efficient and stable supply chain system to ensure product quality and supply efficiency, and the upgrading of digital capabilities helps to alleviate the degree of information asymmetry in companies, enabling companies to obtain more valuable information resources [49]. The use of emerging technologies can enable supply chain members and stakeholders to effectively mitigate any supply chain issues related to non-conforming products based on the level of supply chain data accumulation [50]. This study measures the intelligent supply chain capability of pre-cooked meal companies, based on whether they have implemented intelligent production, intelligent storage, intelligent transportation, and applied emerging technologies, and have achieved the automation and intelligence of supply chain processes. If two conditions are met, it is assigned a value of 1; otherwise, it is assigned a value of 0.

Organizational Dimension: The analysis of the organizational dimension focuses on internal aspects such as strategic planning, brand development, and channel expansion within enterprises. Prepared food companies must optimize their product structure through strategic product planning, sustained R&D investment, and the enhancement of product strength, thereby increasing profit margins. Additionally, the international business team and internal support systems of a company are equally crucial for achieving high-quality development in overseas markets. The establishment of a food safety management system and supply chain partnership relationships reflect the company’s internal management and external collaboration capabilities. Food safety is directly related to brand image and consumer trust, while partnerships form the basis for supply chain collaboration and risk-sharing. Therefore, this study selects the construction of a food safety management system, supply chain partnership relationships, and talent and social adaptability as factors to measure the organizational dimension of the overseas supply chain of pre-cooked food enterprises.

(1) Food Safety Management System

The construction of a food safety management system refers to a set of systems, processes, and technical means that is established by pre-cooked meal companies to ensure food safety [51]. This system should cover the entire process from raw material procurement to product sales, with strict control over key system links to ensure the safety and quality of pre-cooked meals. Food safety is the cornerstone of the development of pre-cooked meal companies. Based on the reporting period, the presence or absence of food safety issues is used to assess whether the construction and implementation of the food safety management system are in compliance. Companies with food safety issues are assigned a value of 0, while those without food safety issues are assigned a value of 1.

(2) Supply Chain Partnership Relationships

We use the supply chain partnership relationship to describe the partnership relationships within the company’s supply chain. Overall supply chain concentration refers to the degree of dependence of the company on its suppliers and customers throughout the supply chain [52]. Good partnership relationships can promote writing in the upstream and downstream areas of the supply chain and reduce resource waste, and, through close cooperation, all parties in the supply chain share social responsibilities and ensure fair trade. This study uses the supplier concentration of pre-cooked meal companies to measure supply chain partnership relationships. By collecting the supplier concentration information provided in the annual reports of various listed companies, supplier concentration is calibrated using the quartile method to measure the variable of supply chain partnerships.

(3) Talent and Social Adaptability

Talent and social adaptability refer to an organization’s capability in terms of human resource management, talent cultivation, social responsibility, and integration with the societal environment [53]. This encompasses the strategies that enterprises employ to attract, develop, and retain talent, as well as their ability to contribute to societal well-being and adapt to societal changes through their business operations. The importance of corporate culture in modern enterprises cannot be overstated, as it serves as a pivotal factor underpinning the healthy development of any organization [54]. As the ready-to-cook food industry continues to expand, in the process of enterprises going global, the need for talented personnel who are adept in diverse political and cultural environments is paramount. Enhancing digitalization efforts and attracting high-caliber, demanding support personnel have become pivotal to team building [55]. This study assesses talent and social adaptability in ready-to-cook food enterprises by examining whether the companies have established a professional talent pool and fostered a distinctive corporate culture. An organization that fulfills either criterion is assigned a value of 0.67, whereas meeting both criteria is awarded a score of 1.

Environmental Dimension: An exploration of the environmental dimension encompasses external factors such as market demand, policy and regulation, and cultural differences. Prepared food enterprises must take into account cultural adaptability, market positioning, product quality, and channel layout during their overseas expansion. Long-term educational and promotional activities targeting the overseas market are indispensable for establishing consumer trust and promoting the sustainable development of the prepared food industry abroad. Furthermore, policy risk assessment and management are key risk factors that Chinese enterprises must consider first during overseas expansion [56]. Market adaptability, compliance, and risk management reflect the enterprise’s sensitivity and response capacity to changes in the external environment. Market adaptability determines whether the enterprise can respond quickly to consumer needs and market trends, while compliance and risk management ensure the enterprise’s legal and compliant operations in the global market, reducing potential legal and reputational risks. Therefore, this study selects market adaptability, compliance and risk management, and product line richness as factors to measure the environmental dimension of the overseas supply chain of pre-cooked food enterprises.

(1) Market Adaptability

Developing appropriate market plans for prepared food enterprises can help clarify market positioning, formulate effective marketing strategies, assess market risks, and guide the development direction of the enterprise, ultimately enhancing its competitiveness and sustainable development capabilities in the market [57]. Strategic adjustments to enhance environmental adaptability and maintain enterprise survival and growth are the norms of modern business operations [58]. This study measures whether enterprises have introduced corresponding market plans by examining whether prepared food enterprises have proposed clear market plans and development directions, as well as whether they have begun to scale in overseas markets or have overseas expansion plans. Based on the 2022 annual reports of 17 enterprises, a binary assignment is made for whether listed companies have clearly planned for the market, assigning a value of 1 to enterprises with clear market plans and plans for overseas development or existing overseas markets, and assigning a value of 0 to enterprises with only domestic market plans.

(2) Compliance and Risk Management

Product compliance refers to a company’s adherence to applicable laws, regulations, industry standards, contractual requirements, and ethical norms [59]. Conversely, risk management involves the process of identifying, assessing, prioritizing, controlling, and mitigating the uncertainties and potential threats that could affect the business. Effective compliance and risk management can help enterprises prevent or reduce potential losses and protect enterprise value. This study assigns a value of 1 to listed prepared food enterprises that have established relevant legal and regulatory systems in their 2022 annual reports, such as providing detailed measures for implementing laws and regulations, and a value of 0 otherwise.

(3) Product Line Richness

Product line richness refers to the diversity and breadth of the products or services offered by an enterprise [60]. A company with a rich product line can provide a variety of different products or services to meet the needs and preferences of different consumers. For a prepared food enterprise, continuous product innovation is a crucial factor in maintaining market share. This study collects data on the number of product lines of prepared food enterprises and measures the variable of product line richness after calibrating the number of product lines using the quartile method.

Based on the selection, analysis, and interpretation of the above variables, this study summarizes the assignment rules for conditional and result variables, as well as the types of variables, into a table, as shown in Table 2. 

This study employs the ESG rating of enterprises as the outcome variable, which serves as a comprehensive indicator for measuring corporate sustainability performance. Corporate sustainability is a vision for long-term development, and ESG offers a practical framework and assessment standard for achieving this vision [61]. Through the practice of ESG, enterprises can ensure that their strategies and operations meet the requirements of sustainable development, thereby gaining advantages in terms of global competition [62]. The TOE theory provides a perspective for analyzing enterprise innovation and transformation from the three dimensions of technology, organization, and the environment, while ESG emphasizes enterprises’ performance regarding environmental, social, and governance aspects. The combination of the two can provide a comprehensive analytical framework that covers multiple aspects of enterprise operations. It considers the behavior of enterprises in actively fulfilling their social obligations, consciously creating value, taking risks, and achieving social fairness and justice while pursuing profit maximization. It is also a manifestation of corporate governance dimensions. Enterprises with market power can promote green innovation by fulfilling their strategic corporate social responsibility [63]. This study evaluates whether 17 listed prepared food enterprises assume the corresponding social responsibilities, based on their ratings in Wind ESG. The ratings are divided into seven standards: AAA, AA, A, BBB, BB, B, and CCC. Among the 235 enterprises participating in the ESG rating, we use AAA as the complete membership cut-off point, BBB as the crossover point, and CCC as the complete non-membership cut-off point for data calibration, adjusting the cooked value of the crossover point to 0.499.

Through this structured TOE theoretical analysis, which integrates the technological, organizational, and environmental dimensions of the TOE theory with the environmental, social, and governance factors of ESG, enterprises can more comprehensively identify and manage the risks related to technological innovation, organizational change, and environmental adaptability. This provides a comprehensive theoretical perspective for the generation mechanism of supply chain sustainability for listed prepared food enterprises expanding into overseas markets and serves as an empirical basis for providing suggestions to enterprises in formulating strategies and to policymakers.

### 3.4. Introduction to fsQCA Analysis Method

The fsQCA method utilizes set theory to identify the common characteristics among different cases and employs fuzzy set theory to handle subtle differences among cases. Fuzzy sets allow for partial membership, meaning that cases can belong to a set to varying degrees. Based on the principle of configurational equivalence, which posits that different combinations of conditions can produce the same result [20], the fsQCA analysis process involves the following key steps:

(1) Variable calibration: Converting raw data into fuzzy set membership scores and establishing thresholds to distinguish the membership status of cases, laying the foundation for subsequent analysis.
(2) Necessity analysis: Identifying condition variables that have a direct impact on the outcome variable and determining the necessary conditions for the occurrence of the outcome.
(3) Sufficiency analysis of conditional configurations: Utilizing logical minimization techniques to identify the minimal combinations of conditions that will produce the outcome, which are logically irreducible.
(4) Robustness testing: Ensuring the reliability and stability of the analysis results through sensitivity analysis and cross-validation.

After completing the above analysis, the fsQCA method will reveal three types of solutions: complex solutions, parsimonious solutions, and intermediate solutions. The complex solution provides all possible configurations, the parsimonious solution highlights the core conditions, and the intermediate solution offers a balanced perspective. In-depth interpretation of the generated solutions involves identifying core and marginal conditions and validating these findings using theoretical frameworks and other evidence.

**Figure 1:**
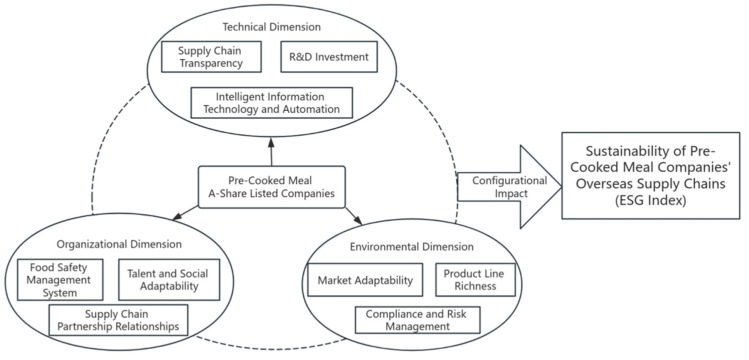
Research model of the sustainable generation mechanism of listed pre-cooked meal companies’ overseas supply chains.

This study focuses on the generation mechanism of supply chain sustainability for Chinese A-share listed pre-prepared food companies that are expanding into overseas markets. Since this issue is multifaceted and complex, fsQCA is an excellent tool for handling such complexity, especially with a limited number of cases and a large number of variables. It possesses characteristics such as interdependence among factors, equivalence of configurational pathways, and causal asymmetry [64]. Given that the subject of this study is listed pre-prepared food companies with a limited sample size, fsQCA provides an effective analytical method. Additionally, fsQCA can not only explore the combined effects of different factors but can also verify and expand the existing theories, ensuring both scientific rigor and accuracy in the analysis, while improving the credibility of the research results through robustness testing. The research model is shown in Figure 1.

## 4. Empirical Analysis

### 4.1. Variable Calibration 

In this study, we employed a binary crisp-set calibration method to assess whether supply chain transparency, intelligent information technology and automation, food safety management system, market adaptability, and compliance and risk management meet their respective set standards. For the remaining influential factors, four-point fuzzy-set calibration was conducted based on the data collected from corporate annual reports. In the absence of theoretical, empirical, or reported news to guide calibration, the 95th and 5th percentiles of the sample data were used as thresholds for full membership and non-membership, respectively, with 50% serving as the crossover point. The fsQCA 4.0 software was utilized to convert the aforementioned data into set membership values.

### 4.2. Necessity Analysis 

Table 3 presents the results of the necessity condition test for higher and lower ESG ratings, analyzed using the fsQCA 4.0 software. The necessity analysis of individual variables is a method used to measure the explanatory power of a single variable among the selected variables on the outcome variable. Consistency assesses the degree of agreement when the evaluation results of a necessary condition are considered essential; coverage evaluates the relevance of the necessary condition, that is, the degree to which instances of the condition correspond with instances of the outcome. The necessity of an individual variable primarily depends on the level of consistency. Conditions such as compliance and risk management and those of talent and social adaptability, with consistencies above 0.9, indicate a high degree of alignment with sustainability. Other conditions fall below the 0.9 threshold for determining necessity. Investment in research and development, supply chain transparency, intelligent information technology and automation, the construction of a food safety management system, supply chain partnership relationships, market adaptability, and product line richness are sufficient conditions for the sustainable development of Chinese prepared food enterprises in overseas markets, playing a promotional role in their international expansion. Since the development of a company’s overseas supply chain is the result of a combination of multiple factors, a configurational analysis of conditions is also warranted.

### 4.3. Configurational Analysis

Configurational analysis primarily reveals the combinations of condition attributes related to the sustainable development of the overseas supply chain of pre-cooked meal companies, with various variable configurations expressing different functionalities, effects, and pathways [65]. The sufficiency of condition combinations is analyzed based on the truth table, establishing thresholds for raw consistency and case frequency to construct the truth table [66]. In this study, when exploring the sufficiency of conditions affecting the sustainable supply chain of pre-cooked meal companies going overseas, the case frequency threshold is set to 1, retaining more than 80% of the total number of cases, with the minimum standard for raw consistency set to 0.78, and the PRI (problem-relevance index) consistency threshold set to 0.75 [67].

The fsQCA analysis yields three types of solutions, with the mechanisms that appear in both intermediate and parsimonious solutions considered to be core influencing factors, while conditions that appear only in the intermediate solution and not in the parsimonious solution are regarded as auxiliary conditions. Therefore, this study chooses to combine intermediate and parsimonious solutions to elaborate on the configurational results [68]. Balanced coverage and consistency are more persuasive in the analysis of results [69]. As can be seen from the table, the solution’s coverage is 0.51281 and the solution’s consistency is 0.891717, indicating that the model in this study has good explanatory power. The coverage of 0.51281 means that the following condition configurations can explain 51.281% of the cases. Moreover, both the coverage and consistency of the solution are above the critical values, indicating that the analysis results are valid.

The fsQCA analysis results reveal that there are five condition configurations for the mechanism of sustainability generation of the overseas supply chain of pre-cooked meal companies. This study categorizes configurations with the same core conditions into a single pattern and divides them into three distinct pathways, according to the utility needs reflected by the core conditions: the technology-driven and norm compliance model, the market sensitivity and product innovation model, and the robust operation and risk control model. The specific details are presented in Table 4.

### 4.4. Multidimensional Analysis of Sustainability Generation Pathways

(1) Path 1: Technology-Driven and Norm Compliance Model

The core conditions of this pathway are R&D investment (RDI), intelligent information technology and automation (IITZ), and compliance and risk management (CRM). Sustained R&D can drive product innovation and process improvement, utilizing information technology to enhance the efficiency and transparency of the supply chain. Compliance ensures that enterprises adhere to international trade regulations and manage potential risks. The auxiliary conditions are talent and social adaptability (TSA) and market adaptability (~MA), ensuring that enterprises have personnel capable of adapting to social changes and market demands. Although market adaptability is not included in all pathways, it is crucial for meeting the demands of different markets. This pathway is suitable for technology-driven enterprises with strong R&D capabilities, especially those focusing on enhancing product competitiveness through technological innovation, with target markets that have high standards for food safety, quality, and technology.

(2) Path 2: Market Sensitivity and Product Innovation Model

The core conditions of this pathway are market adaptability (MA) and product line richness (PLR). Firstly, products and strategies must adapt to the specific needs of the target market. Secondly, enterprises also need to offer a diverse range of products to meet the needs of different consumers. The auxiliary conditions are R&D investment (~RDI) and supply chain partnership relationships (~SCP). Although R&D investment is not a core element in this pathway, it is also a key factor in supporting product innovation and diversification. Moreover, having a good partnership is conducive to better adaptation to market changes. This pathway is suitable for market-sensitive enterprises with a rich product line, especially those companies that can quickly respond to market changes and adjust their product strategies, with target markets having diverse consumer demands and a broad need for product variety and different flavors.

(3) Path 3: Robust Operation and Risk Control Model

The core conditions of this pathway are the establishment of a food safety management system (FSMS) and compliance and risk management (CRM) to ensure the quality and safety of food, which are essential for products to meet the high standards of the international market. Compliance is a crucial part of the foundation, along with the technology and compliance-driven pathway. The auxiliary conditions are supply chain transparency (~SCT) and supply chain partnership relationships (SCP); transparency in the supply chain aids in risk management and builds consumer trust, and partnerships may further strengthen the food safety system and risk management. This pathway is suitable for risk-averse enterprises that focus on long-term stable development, especially companies with advantages in terms of food safety and quality control, targeting markets with strict food safety and quality requirements, regulations, and complex or uncertain market environments.

### 4.5. Robustness Test 

There are numerous methods by which to test the robustness of qualitative comparative analysis (QCA), a common approach being the adjustment of calibration references and consistency threshold values. By comparing the analytical outcomes before and after parameter adjustments, one can assess whether there are any substantial changes. The absence of such changes indicates that the results are robust, as noted by Leppänen [70] and Shi and Wang [44]. Furthermore, this study employs the two criteria proposed by Schneider and Wagemann [71] for assessing the robustness of QCA results: the set-theoretic relationship among different configurations and the differences in fit parameters across configurations. By adjusting the consistency threshold and the minimum number of cases, it was observed that there were no changes in the configuration paths, core variables, auxiliary variables, and indicators such as consistency and coverage rates. Ultimately, the findings of this study were found to remain robust.

## 5. Discussion

In the tide of global integration, China’s prepared food industry has rapidly secured a significant position in the food supply chain domain, leveraging its convenience and diverse product options. As market competition intensifies, constructing a sustainable supply chain for prepared foods going global is crucial for companies to achieve their internationalization strategies. However, existing research primarily focuses on the industry and consumer perspectives [72], leaving gaps in understanding the sustainability mechanisms, key success factors, and how to optimize supply chain management to enhance the overseas market sustainability performance of prepared food enterprises going global. To address these underexplored issues, this study adopts an innovative research methodology. By employing the TOE framework and applying fuzzy-set qualitative comparative analysis (fsQCA), an in-depth analysis of 388 data points from 17 listed Chinese prepared food companies was conducted. This study aims to fill the research gaps and provide theoretical foundations and practical guidance to ensure the sustainability of the supply chains of companies that are going global.

From the perspective of research subjects and content, although scholars such as Henrique Espírito Santo and Belem Barbosa [73], Miria Lazaris and Susan Freeman [74], and Kevin I. N. Ibeh et al. [75] have studied market opportunities, key success factors, and successful strategies in the internationalization process of small- and medium-sized enterprises (SMEs), there is a current dearth of research on large enterprises, especially concerning the sustainability mechanisms of supply chains for listed companies in the burgeoning trend of prepared food enterprises going global. As an emerging and promising field of research, internationalization research on SMEs may not be applicable to large enterprises. Therefore, companies still need to explore the path to sustainable supply chains becoming global, and this study aims to complement and expand the existing literature.

From a theoretical standpoint, the existing studies seldom utilize theoretical frameworks, whereas this study combines the TOE framework with the concept of Environmental, Social, and Governance (ESG), offering a novel theoretical perspective on the sustainability of supply chains for prepared food enterprises going global. This integration not only spans multiple aspects of corporate operations but also provides theoretical support for understanding corporate supply chain management in the context of globalization. Additionally, this study constructs an indicator system regarding the factors influencing the sustainability of supply chains for prepared food enterprises that are going global, helping stakeholders identify key determinants of supply chain sustainability and offering theoretical support for the supply chain management of listed companies in the international market.

In terms of variable selection, this study extends and expands the variables proposed by Yao [76], Huang and Tang [39], and Chen and Duan [40]. It includes not only key elements from traditional food supply chain research, such as the perfection of credit management mechanisms, innovation risk reduction mechanisms, strict qualification review of foreign merchants, and data protection management mechanisms (Song and Li [77]), but also emphasizes modern supply chain key elements such as R&D investment, supply chain transparency, intelligent information technology and automation, the establishment of food safety systems, and acceptance in different cultural and political environments. These factors comprehensively cover the main needs for the sustainable development of supply chains in the process of going global for prepared food enterprises. The introduction of these new variables or measurement methods helps to more fully understand the sustainability of supply chains in terms of the process of prepared food enterprises that are going global and provides new research perspectives and analytical methods on the existing literature.

Regarding the analytical approach, unlike previous studies that focus on single-dimensional analysis, this study comprehensively considers the complexity of multiple factors interacting with each other. While existing studies often employ qualitative analysis or macro-analysis methods, such as the CAGE framework [78], this study combines quantitative and qualitative methods, using the TOE framework and fsQCA analysis to focus on the key factors affecting the overseas supply chains of listed prepared food companies in China, offering a new perspective for optimizing supply chain management and helping to enhance the sustainability performance of companies going global (Yang and Xu [27]; Zhao [79]). This multidimensional analytical method not only reveals the key factors affecting the sustainability of overseas supply chains of prepared food enterprises but also identifies the successful paths that support Chinese listed prepared food companies in the overseas market, according to various factor combinations.

In summary, the research findings not only provide theoretical support for sustainable supply chain management of A-share listed companies in the international market but also offer practical strategic guidance for their expansion in overseas markets, while simultaneously offering support for scientific decision-making by policymakers. Specific recommendations are as follows:

(1) Recommendations for Corporate Decision-Making

① For the technology and compliance-driven pathway, companies should continue to invest in R&D to innovate products and optimize processes, thereby enhancing their technological competitiveness. Utilizing intelligent information technology and automation can improve the efficiency of the supply chain and increase transparency, allowing companies to better track product flow and increase consumer trust. Strengthening the compliance system ensures that companies adhere to regulations in global trade, reducing legal and operational risks. Emphasizing talent cultivation and team building ensures that the company has personnel who are capable of adapting to social changes and market demands. Although market adaptability is an auxiliary condition, companies still need to pay attention to market adaptability and flexibly adjust their strategies to meet the needs of different markets.

In the pursuit of sustainable and international supply chain development, enterprises must regard technological innovation and compliance as core drivers. Companies should establish a comprehensive set of supply chain policies that clearly articulate their commitment to technological advancement and social responsibility, ensuring these policies are implemented at all organizational levels. Through open and transparent communication channels, such as corporate websites and regular reports, they should convey their commitments and practices regarding sustainable supply chains to all stakeholders. Concurrently, companies need to establish robust internal monitoring and coordination mechanisms to ensure that supply chain policies align with business objectives and to resolve potential inter-departmental conflicts. Furthermore, enterprises should invest their resources in R&D activities to foster technological innovation, thereby enhancing the efficiency and responsiveness of supply chains while ensuring that technological applications adhere to ethical and social responsibility standards. Compliance is key to a company’s internationalization strategy; thus, companies should institute stringent compliance oversight systems to ensure adherence to international laws, regulations, and industry standards by both them and their supply chain partners. This not only helps mitigate legal risks but also reflects the company’s commitment to corporate social responsibility. By adopting these measures, companies will be able to build an advanced and compliant supply chain system, providing a solid foundation for long-term development and competitiveness in the global market.

② For the market adaptability and product diversity pathway, enterprises should closely monitor market dynamics, guide product lines and strategies based on market and consumer demand, and respond quickly to market changes. Offering a diverse range of products can meet the tastes and needs of different consumers and enhance market competitiveness. Even if R&D investment is not a core condition of market growth, it should be regarded as a key factor in supporting product innovation and diversification. At the same time, it is important to establish and maintain good supply-chain partnership relationships to improve market adaptability and flexibility, and to build mechanisms that can respond quickly to market changes, including flexible production scheduling and inventory management.

For companies to achieve success in the fiercely competitive global market, they must make market adaptability and product diversity the cornerstone of their supply chain strategy. Enterprises need to conduct in-depth market research to understand the needs and preferences of consumers in different regions and transform these insights into an impetus for innovation to develop products that cater to local markets. This market-oriented product development strategy requires companies to have the ability to quickly respond to market changes and flexibly adjust production lines to meet diverse consumer demands. Concurrently, companies should enhance their collaboration with supply chain partners by providing the necessary technical support and building capacity, helping their partners to improve their production capabilities and product quality. Such cooperation not only strengthens the overall competitiveness of the supply chain but also promotes joint innovation among upstream and downstream stakeholders. Furthermore, companies should strive to improve the transparency of the supply chain by publicly disclosing basic information about supply chain partners, responsible management practices, and the effectiveness of supervisory measures, thereby enhancing the trust of consumers and stakeholders. Transparent supply chain management is not only conducive to establishing a responsible brand image for the enterprise but is also an essential pathway toward enhancing the sustainability of the supply chain.

③ For the infrastructure and risk management pathway, enterprises should establish stringent food safety management systems to ensure product quality and safety, meeting the high standards of the international market. A comprehensive risk management mechanism should be established, including risk identification, assessment, mitigation, and monitoring. Further enhancing the transparency of the supply chain means that consumers and partners can clearly understand product information, increasing trust. Strengthening partnerships with upstream and downstream supply chain partners will jointly address risks and improve the overall stability of the supply chain. It is also important to focus on long-term stable development, avoid short-term actions, and ensure the sustainable development of the enterprise.

In constructing a foundation for infrastructure and risk management, companies must implement a series of comprehensive measures to ensure the robustness and adaptability of their supply chains. Initially, enterprises should establish a comprehensive supply chain risk assessment system to identify potential risk points, such as supplier dependency and logistics disruptions, and develop corresponding response strategies. This includes formulating detailed crisis management plans and enhancing supply chain transparency and flexibility by optimizing supply chain structures, reducing intermediary links, and establishing direct dialog and cooperation mechanisms.

Concurrently, companies need to implement due diligence processes to ensure that supply chain social responsibilities and environmental standards are met. This involves not only the supervision and evaluation of supply chain partners but also the continuous improvement of the company’s own operations. Enterprises should disclose their due diligence measures and performance transparently to stakeholders to enhance trust and brand reputation.

Furthermore, companies should strengthen collaboration with supply chain partners to jointly develop new technologies and solutions, thereby enhancing the sustainability and risk resistance of the supply chain. This may involve leveraging technologies such as the Internet of Things, big data analysis, and artificial intelligence to optimize inventory management, demand forecasting, and risk early warning systems. Additionally, companies should establish performance monitoring systems to regularly assess the efficiency, cost-effectiveness, and sustainability performance of their supply chains, making continuous improvements based on the assessment results. This data-driven decision-making process helps enterprises to adjust their strategies in a timely manner in response to the ever-changing market and environmental conditions.

(2) Recommendations for Policymakers

① Deepening policy support and developing incentive mechanisms: Policymakers should tailor specialized policies to meet the specific needs of the prepared food industry from a global perspective, adapting to the political, cultural, and legal environments of different countries. For instance, additional R&D funding could be provided to companies that adopt new cold-chain logistics technologies, as well as offering support to help meet the carbon emission policies of some European nations. Promoting the establishment of industry standards and offering certification and rewards to prepared food export enterprises that meet these standards can enhance their competitiveness in the international market. This support will help these enterprises to better develop sustainable supply chains in environments with heterogeneous regulatory frameworks.

② International cooperation and trade facilitation: The Chinese government should actively establish trade dialog mechanisms with major export market countries, and, in light of the particularities of the pre-cooked meal industry, should promote the development of internationally recognized product standards and certification systems. Through diplomatic channels, they should provide legal and commercial support for Chinese pre-cooked meal enterprises in overseas markets to reduce the uncertainty of cross-border operations.

③ Professionalization of talent cultivation and educational collaboration: Collaboration between renowned domestic and international universities could be developed to establish a specialty in a supply chain management qualification for the pre-cooked meal industry, focusing on nurturing talents with a global perspective and professional supply chain management skills. Enterprises should be encouraged to partner with academic institutions to initiate supply chain innovation projects, providing students with practical operational experience while also bringing new solutions to the companies.

④ Precision in market information platform construction: The establishment of a market information platform is an important step for the pre-cooked meal industry. It should integrate multi-dimensional data such as international market demands, consumer preferences, and competitor dynamics, offering companies precise market insights. The platform should be equipped with data analysis capabilities, utilizing technologies like machine learning to forecast market trends and guide product development and marketing strategies for businesses.

## 6. Conclusions and Future Prospects

### 6.1. Conclusions

In the wave of global integration, China’s pre-cooked meal industry, with its convenience and diverse choices, has rapidly become an important participant in the field of food supply chains. Amid increasingly fierce competition in both domestic and international markets, supply chain sustainability has become a core element of corporate internationalization strategy. This study focuses on the sustainability generation mechanism of the overseas supply chain of Chinese pre-cooked meal A-share listed companies, combining the TOE theoretical framework with environmental, social, and governance factors from ESG and the fuzzy-set qualitative comparative analysis (fsQCA) method. An in-depth analysis of 388 data items from 17 listed companies was conducted, leading to the following conclusions:

(1) Construction of a sustainability generation mechanism indicator system for pre-cooked meal manufacturers’ overseas supply chains: The study constructs a model of factors affecting the sustainable development of pre-cooked meal companies going overseas, based on the three dimensions of environment, organization, and technology. This model comprehensively reflects the key elements that promote corporate sustainable development in the current development context.

(2) Identification of key paths in the sustainability generation mechanism for pre-cooked meal manufacturers’ overseas supply chains: The research results reveal that talent and social adaptability, compliance, and risk management are important factors in promoting sustainable development in terms of the market for pre-cooked meal companies overseas. The empirical analysis in the model indicates that the constructed configuration paths not only fit the current development status of enterprises but also have good explanatory power, as follows:

① Technology-driven and norm compliance model: This model emphasizes the importance of scientific and technological research and development and of technological innovation capabilities, an area that is suitable for enterprises with a distinct advantage in the field of technology. ② Market sensitivity and product innovation model: This is suitable for companies that can quickly respond to market changes and flexibly adjust their product strategies. With this model, product research and development innovation are key to the sustainable development of corporate overseas supply chains. ③ Robust operation and risk control model: The coverage rate of this model is significantly higher than that of other models, indicating its greater strength for explaining the sustainable development of pre-cooked meal manufacturers overseas.

(3) Strategic recommendations: Based on the analysis results derived from the TOE theory and the environmental, social, and governance factors in ESG, along with the fsQCA method, listed companies need to focus on technological innovation and intelligentization to enhance supply chain efficiency and transparency while ensuring compliance, cultivating talent with market adaptability, establishing food safety and risk management systems, and promoting sustainable development. Policymakers should formulate supportive policies to encourage the adoption of technology and the establishment of standards, strengthen international cooperation to reduce the risks of overseas operations for businesses, collaborate with educational institutions to cultivate professional talent, and establish market information platforms to drive industry insights with data.

Through these integrated measures, companies will be able to respond flexibly to market changes, while the government can effectively support industry development, jointly promoting international competitiveness and the sustainable development of the pre-cooked meal industry.

### 6.2. Future Prospects

Although this study utilized qualitative comparative analysis and fsQCA methods to conduct an in-depth analysis of the 2022 annual reports of Chinese pre-cooked meal companies, yielding valuable preliminary results, there are still several limitations that point to directions for future research.

Firstly, due to limitations in data availability and the impact of the COVID-19 pandemic, the time span of this study is relatively short. Future research should expand the time frame to cover data over more years, which will not only enhance the robustness of the results but also reveal long-term trends and cyclical changes in industry development, laying a more solid foundation for theoretical construction.

Secondly, the solution coverage of this study is 0.51281, which suggests that the explanatory power of the model requires enhancement. Future research should expand the sample size, particularly by including companies that are active in overseas markets, to improve the coverage and external validity of the study. Moreover, a more in-depth analysis of conditional variables that may affect the model’s explanatory power is needed. These should include market dynamics, consumer behavior, and the development of new food processing technologies to further optimize the model.

Lastly, considering that this study focuses on A-share listed companies producing pre-cooked food in China, future research should extend the sample scope to pre-cooked food enterprises in other regions or countries for comparative analysis. This will verify the universality and applicability of the conclusions drawn from this study. Additionally, conducting categorized research on different types of pre-cooked food enterprises, such as specialized pre-cooked food companies and catering supply-chain enterprises, will help provide more customized sustainability development strategies for these enterprises and summarize the successful overseas expansion experiences of pre-cooked food companies.

Through future research in these directions, we can gain a more comprehensive understanding of the drivers of high-quality development for pre-cooked food enterprises and provide richer theoretical support and practical guidance for the healthy development of the global pre-cooked food industry.

## Figures and Tables

**Table 1:** Types of prepared food enterprises and their representative companies.

Type of Prepared Food Enterprise	Representative Companies
Specialized Prepared Food Enterprises	Wei Zhi Xiang, Gai Shi Food
Catering Supply Chain Enterprises	Sheng Nong Development, Long Da Food, Guo Lian Aquatic Products, Qian Wei Central Kitchen, Chun Xue Food, Fu Cheng Shares, Yi Ke Food,
Frozen Food Enterprises	An Jo Food, San Quan Food, De Li Si, Hai Xin Food, Hua Ying Agriculture, Hui Fa Food, Shuang Hui Development
Catering Enterprises	Guangzhou Restaurant

Data source: China Media Group’s “Top 50 Leading Prepared Food Enterprises of 2023” list.

**Table 2:** Conditional variable, Measurement Criteria, variable types, and assignment descriptions.

Variable Name	Measurement Criteria	Valuation Rules	Variable Type
R&D Investment	R&D Investment Data from Corporate Annual Reports	Calibrated using the quartile method, ultimately scaled to a 0–1 range.	Continuous Variable
Supply Chain Transparency	Presence of a Supply Chain Traceability System	Present = 1, Absent = 0	Discrete Variable
Intelligent Information Technology and Automation	Engagement in Intelligent Production, Intelligent Storage, Intelligent Transportation; Application of Emerging Technologies; Achievement of Automation and Intelligent Supply Chain Processes	1 if meeting two or more criteria, otherwise 0	Discrete Variable
Food Safety Management System	Occurrence of Food Safety Issues During the Reporting Period	Yes = 0, No = 1	Discrete Variable
Supply Chain Partnership Relationships	Supplier Concentration in Prepared Food Enterprises	Calibrated using the quartile method, ultimately scaled to a 0–1 range	Continuous Variable
Talent and Social Adaptability	Professional Talent Development and Corporate Culture Formation	0.67 for meeting one criterion, 1 for meeting both	Continuous Variable
Market Adaptability	Clear Market Planning and Development Direction; Scale or Plans for the Overseas Market	1 for companies with clear market plans and overseas development plans or presence, 0 for companies with domestic market plans only	Discrete Variable
Compliance and Risk Management	Establishment of Legal and Regulatory Systems	1 for presence, 0 for absence	Discrete Variable
Product Line Richness	Product Line Richness of Prepared Food Enterprises	Calibrated using the quartile method, ultimately scaled to a 0–1 range	Continuous Variable

**Table 3:** Necessity analysis.

Antecedent Variables		Sustainability	Sustainability
		Consistency	Coverage
Technical Dimension	R&D Investment	0.456080	0.854476
	~R&D Investment	0.740037	0.619489
	Supply Chain Transparency	0.649756	0.581000
	~Supply Chain Transparency	0.350244	0.574167
	Intelligent Information Technology and Automation	0.198048	0.649333
	~Intelligent Information Technology and Automation	0.801952	0.563429
Organizational Dimension	Food Safety Management System	0.502440	0.549111
	~Food Safety Management System	0.497560	0.611750
	Supply Chain Partnership Relationships	0.548190	0.628438
	~Supply Chain Partnership Relationships	0.564559	0.659501
	Talent and Social Adaptability	0.943066	0.645961
	~Talent and Social Adaptability	0.268402	1.000000
Environmental Dimension	Market Adaptability	0.594144	0.649333
	~Market Adaptability	0.405856	0.499000
	Compliance and Risk Management	1.000000	0.578588
	~Compliance and Risk Management	0.000000	0.000000
	Product Line Richness	0.522774	0.673037
	~Product Line Richness	0.655958	0.689316

**Table 4:** Configurations of sustainability generation mechanisms for the overseas supply chains of pre-cooked meal enterprises.

Conditional Variables	S1	S2	S3	S4	S5
R&D Investment (RDI)	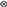	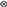	●	●	●
Supply Chain Transparency (SCT)	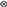	•	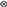	•	•
Intelligent Information Technology and Automation (IITA)	⊗	●	●	⊗	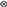
Food Safety Management System (FSMS)	●	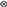	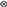	●	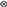
Supply Chain Partnership Relationships (SCP)	⊗	•	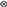	⊗	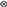
Talent and Social Adaptability (TSA)	●	●	●	●	●
Market Adaptability (MA)	⊗	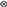	•	⊗	•
Compliance and Risk Management (CRM)	●	●	●	●	●
Product Line Richness (PLR)	●	⊗	⊗	●	●
Raw Coverage	0.156263	0.0558154	0.0681171	0.0578487	0.116816
Unique Coverage	0.152196	0.0558154	0.0681171	0.053782	0.114782
Solution Consistency	0.891717				
Solution Coverage	0.51281				

Please note that ● denotes the presence of a core condition. • Indicates the presence of a secondary condition. ⊗ Signifies the absence of a core condition. 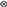 Shows the absence of a secondary condition.

## Data Availability

The original contributions presented in the study are included in the article, further inquiries can be directed to the corresponding author.

## List of Abbreviations

TOE	Technology-Organization-Environment Framework
ESG	Environmental, Social, and Governance Criteria
RDI	Research and Development Investment
SCT	Supply Chain Transparency
IITA	Intelligent Information Technology and Automation
FSMS	Food Safety Management System
SCP	Supply Chain Partnership Relationships
TSA	Talent and Social Adaptability
MA	Market Adaptability
CRM	Compliance and Risk Management
PLR	Product Line Richness
fsQca	Fuzzy-set Qualitative Comparative Analysis
